# Inclination Changes in Incisors During Orthodontic Treatment with Passive Self-Ligating Brackets

**DOI:** 10.3390/jcm14103370

**Published:** 2025-05-12

**Authors:** Corinna L. Seidel, Uwe Baumert, Franziska Ost, Hisham Sabbagh, Andrea Wichelhaus

**Affiliations:** Department of Orthodontics and Dentofacial Orthopedics, University Hospital, LMU Munich, Goethestrasse 70, 80366 Munich, Germany; uwe.baumert@med.uni-muenchen.de (U.B.); franziska.ost@outlook.de (F.O.); hisham.sabbagh@med.uni-muenchen.de (H.S.); kfo.sekretariat@med.uni-muenchen.de (A.W.)

**Keywords:** orthodontic treatment, self-ligating brackets, passive SL brackets, incisor inclination, proclination, protrusion, cephalometrics, sex-specific inclination change

## Abstract

**Background**: Correct inclination of the incisors is crucial for function and occlusion. The aim of this retrospective cephalometric study was to investigate the effect of orthodontic treatment with passive self-ligating (SL) brackets on the incisor inclination compared to untreated controls. **Methods**: This study evaluated an orthodontic treatment group (*n* = 30, Ø13.4 years, ANB Ø2.3°) before (T1) and after (T2) orthodontic therapy with passive SL-brackets (MBT prescription, 0.022″ slot) by one experienced orthodontist. The control group was matched according to age and sex (*n* = 30, Ø13.3 years, ANB Ø2.5°). The incisor inclination was evaluated digitally using cephalometric lateral radiographs. **Results**: The cephalometric evaluation showed a significant incisor proclination during orthodontic treatment with SL brackets. The upper incisors proclined by +3.8° (U1–PP) and +3.7° (U1–SN) and the lower incisors proclined by +4.4° (L1–MP). The control group presented a reclination of the upper incisors by −1.4° (U1–PP) and −0.6° (U1–SN) and the lower incisors by −0.4° (L1–MP). The clinically relevant protrusion effect during orthodontic treatment with SL brackets summed up to +5.2° (U1–PP), and +4.3° (U1–SN) for the upper incisors (+2.0°/year, +1.6°/year) as well as +4.8° (L1–MP) for the lower incisors (+1.6°/year) compared to controls. Sex-specific differences were found. Males showed a greater proclination of the upper incisors by +5.3° (U1–PP) and +5.9° (U1–SN) compared to females in the treatment group. **Conclusions**: Orthodontic treatment with SL brackets results in protrusion of the upper and lower incisors by +5° compared to controls. These findings must be considered in orthodontic treatment planning according to initial diagnostics.

## 1. Introduction

Ortho-axially inclined incisors in the upper and lower jaw are necessary to achieve a functional, neutral occlusion and an ideal vertical and horizontal overbite [1]. For example, retruded upper front teeth impede the correction of a distal occlusion [1]. Similarly, protruded lower front teeth hinder the correction of a class II malocclusion [1]. An undisturbed function during protrusion and lateral movements of the jaw also requires an ideal inclination of the front teeth [2]. Long-term stability is the main goal of orthodontic treatment. Yet, it is affected negatively if incisors are protruded [3,4,5,6]. The proclination of upper front teeth correlates with a higher risk for trauma of the front teeth [7,8]. Early orthodontic correction of class II and prominent upper incisors with functional appliances reduces the risk of dental trauma [9]. During leveling and aligning with initial archwires, the desired tooth movement can be accompanied by undesired side effects. Protruding moments can be a side effect of vertical leveling and the intrusion of incisors [10,11].

Self-ligating (SL) brackets are used by many orthodontists in daily practice as the insertion and removal of initial leveling archwires is quicker compared to conventional brackets [12]. Yet, the removal of thicker archwires, e.g., 0.019″ × 0.025″ stainless steel (SS), is associated with more pain using SL brackets [12]. The first SL bracket (Russel Lock) was developed by Stolzenberg [13]. Different adaptations of SL brackets were also introduced, e.g., Edgelock^®^ [14], SPEED^®^ [15], Damon^®^ [16,17], and In-Ovation C^®^/In-Ovation R^®^ [18]. They are divided into active and passive SL systems regarding the lid closure for archwire ligation [19]. Active systems use a flexible spring clip that exerts forces on the archwire [12,20]. Passive systems use an additional slide, that is, not exerting forces on the archwire [16,21,22]. They aim to reduce friction, thereby allowing easier sliding of the archwire and reducing the applied force [16,21]. In general, friction depends on several factors and can be reduced or enhanced by modifications of the orthodontic archwire, bracket, and ligation [23,24,25]. Compared to conventional brackets, friction is reduced using passive SL brackets during initial alignment with round leveling archwires [26,27,28]. When using archwires of higher dimensions, e.g., a 0.019″ × 0.025″ SS in a 0.022″ slot system, active SL systems enhance friction compared to passive SL brackets [29,30]. Passive SL present reduced friction values compared to conventional brackets [26,28,29]. Initial alignment is faster using active SL brackets compared to passive SL brackets [12,31]. However, overall, the treatment duration is not significantly different between active and passive systems [12]. There is no difference between SL brackets and conventional brackets regarding the number of appointments [32]. Further, no differences can be observed regarding the rate of orthodontically induced inflammatory root resorptions and the need for extraction therapy [32]. Using cone–beam computer tomography scans, a significant proclination of the lower incisors was seen after orthodontic treatment with active and passive SL brackets without differences between the systems [33]. Cephalometric measurements also revealed that passive SL brackets led to an increase in proclination of the lower central incisors [34,35,36]. While most studies focused on the lower incisors, an investigation of inclination change in the upper and lower jaw revealed a significant increase for both measurements after orthodontic treatment with SL brackets [37]. Further, all these studies using lateral cephalometric radiographs and CDCT images lack a comparison with non-treated controls and sex-specific evaluations. Growth-related changes might lead to a change in incisor inclination. To examine the total amount of inclination changes during orthodontic treatment, growth-related changes must be considered.

The aim of this study was to investigate the inclination of the incisors before and after orthodontic treatment with SL brackets using cephalometrics. Further, the study aimed to compare their cephalometric results with an untreated control group to reveal possible growth-related changes and distinguish them from the effects of the SL brackets. Another goal was to evaluate whether sex has an impact on inclination changes.

## 2. Materials and Methods

This retrospective study was approved by the local ethics committee of the University Hospital Munich, LMU Munich (Munich, vote number 20-1057, date of approval 18 December 2020), prior to the beginning of the study. The study included patients treated in the Department for Orthodontics and Orofacial Orthopedics, LMU Munich, between 1 January 2009 and 29 November 2020. Patient selection was based on predefined inclusion and exclusion criteria. All patients meeting the following inclusion criteria were included: (I) fully toothed upper and lower jaw in permanent dentition (except wisdom teeth); (II) complete orthodontic treatment records consisting of an orthopantomogram, lateral cephalometric radiographs, and diagnostic models (alginate impressions); (III) skeletal class I at the start of orthodontic treatment (cephalometrics: ANB between 0° and 4°); (IV) application of SL brackets (MBT prescription); and (V) buccal multibracket application in the upper and lower jaw. The exclusion criteria were as follows: (I) syndromes; (II) missing teeth (except wisdom teeth); (III) displaced teeth; (IV) extraction cases; (V) combined orthodontic and oral surgery treatments; (VI) dental implants; (VII) diseases of the temporomandibular joint; (VIII) use of second-order bends (flattening of the curve of Spee) or third-order bends (torque); and (IX) skeletal class II or III at the beginning of orthodontic treatment. All patients were treated by one experienced orthodontist (A.W.) to ensure a comparable treatment concept. A defined sequence of orthodontic archwires (016″ Nickel Titanium (NiTi), 0.016″ × 0.022″ NiTi, 0.016″ × 0.022″ stainless steel (SS), 0.018″ × 0.025″ SS, 0.016″ × 0.022″ SS) was applied. A 0.018″ × 0.025″ stainless-steel (SS) archwire was applied for at least 8 weeks during orthodontic treatment. All patients showed only minor anterior crowding or gaps in the upper and lower arches. In the upper arch, anterior space analyses revealed an average of 0.43 mm ± 1.34 mm in the upper arch and −0.01 ± 0.54 mm in the lower arch. The Little irregularity index [38] revealed minimal crowding of 2.32 ± 1.60 in the lower arch and 3.44 ± 2.04 in the upper arch. No interproximal enamel reduction was performed in any of the patients. Patients with functional orthodontic pre-treatment prior to orthodontic treatment were not excluded.

### 2.1. Cephalometric Measurements

**Figure 1:**
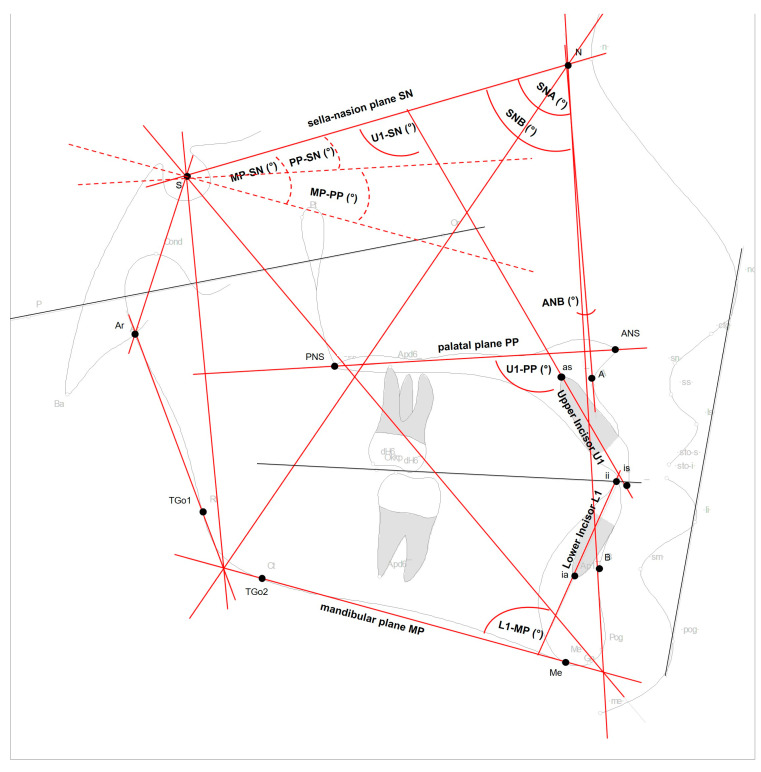
Graphical representation of cephalometric points, planes, and angle measurements used for cephalometric measurements. Red lines indicate cephalometric planes used in this study. Grey lines and points indicate cephalometric planes and points that are included in the cephalometric analyses, but were not analyzed in this study.

Regarding the treatment group, cephalometric lateral radiographs were taken of each patient at two different time points: before bonding the brackets at the start of orthodontic treatment (T1) and after or shortly before removal of the brackets, i.e., at least one year after insertion of the brackets (T2). The ‘CS 9000 Extraoral Imaging System’ (Kodak, Rochester, NY, USA), which has a focus-to-film distance of 1.5 to 4.0 m, was used to take the cephalometric lateral radiographs. The diagnostic software “FR win professional” (Computer Konkret AG, Falkenstein, Germany; software version 07.01.0002) was used to analyze the digital images. The examiners placed defined cephalometric points in accordance with standard orthodontic cephalometrics [39]: Nasion (N), Sella (S), Basion (Ba), A-point (A), B-point (B), Anterior nasal spine (ANS), Posterior Nasal Spine (PNS), Menton (Me), Articulare (Ar), tangent gonion point 1 (TGo1), tangent gonion point 2 (TGo2), incision superius (is), apex superius (as), incision inferius (ii), and apicale inferius (ai) (Figure 1). The following planes were used: (I) Sella–Nasion plane (SN), the connecting line between point S and point N; (II) palatal plane (PP), the connecting line between ANS and PNS; (III) mandibular plane (MP), the connecting line between Me and TGo2; (IV) upper incisor (U1), the connecting line between is and as; and (V) lower incisor (L1), the connecting line between ii and ia (Figure 1). Cephalometric angle measurements were performed as follows: (I) SNA (°): the angle between S–N and point A; (II) SNB (°): the angle between S–N and point B; (III) ANB (°): the angle between points A, N, and B; (IV) MP–PP (°): the angle between planes MP and PP; (V) MP–SN (°): the angle between planes MP and SN; (VI) PP–SN (°): the angle between planes PP and SN; (VII) U1–SN (°): the angle between U1 and SN; (VIII) U1–PP (°): the angle between U1 and PP; and (IX) L1–MP (°): the angle between L1 and MP.

The control group consisted of orthodontically untreated girls and boys from growth studies at the Universities of Iowa, Oregon, and Denver, which are part of the ‘Craniofacial Growth Legacy Collections Project’ [40]. Each patient in the treatment group was matched with an untreated study participant according to age (±six months) and sex. All untreated study participants of the control group were skeletal class I (ANB 0–4°) at T1. The University of Oklahoma Denver Growth Study included a total of 303 orthodontically untreated girls and boys between 1927 and 1967 who received at least 4 (on average, 11.5) cephalometric radiographs (G. Fräns Currier; Department of Orthodontics—Dental School, University of Oklahoma). The Iowa Facial Growth Study was conducted between 1946 and 1960 and included 92 boys and 91 girls who received cephalometric radiographs quarterly until the age of 5, semi-annually between 5 and 12 years of age and annually until the age of 18 years (Shankar Rengasamy Venugopalan; Department of Orthodontics—College of Dentistry, University of Iowa). The Oregon Growth Study included 357 girls and boys between 3 and 18 years of age receiving lateral cephalometric radiography every six months annually between the late 1950s and the mid-1970s (Steve Duckworth; Department of Orthodontics—School of Dentistry, Oregon Health and Science University).

### 2.2. Statistics

The “IBM SPSS Statistics” program Version 28 (IBM Corp., Armonk, NY, USA) was used for the statistical analysis. For the descriptive statistics of interval-scaled data, the mean, standard deviation, median, minimum, and maximum were calculated. Nominal-scaled data were described with percentages and number/total number. Each dataset was first tested for normal distribution using the Shapiro–Wilk test. If a normal distribution was given, parametric methods (*T*-test for two independent or paired variables) were used. Non-parametric methods (Mann–Whitney U-test for two independent or paired variables) were utilized for variables that did not follow a normal distribution. The significance levels were set as follows: *p* ≥ 0.05 = not significant (n.s.); *p* < 0.05 = significant (*); *p* ≤ 0.01 = very significant (**); *p* ≤ 0.001 = highly significant (***). To evaluate intrarater accuracy (reliability), 10 cephalometric radiographs were analyzed twice by the same examiner at an interval of at least six weeks. The relative technical error of measurement (rTEM) was calculated [41] and accepted as rTEM < 15%. The rTEM for all measured variables was below 5% except for the variable ‘ANB angle’, which presented an rTEM of 13.94%.

A post hoc power analysis was applied for two-sample tests regarding the study and the control group using G*Power (version 3.1.9.6, Mac) [42]. Outcomes concerning matched pairs (study vs. control group, or T2 vs. T1) were determined using the ‘t tests—Means: Differences between two dependent means (matched pairs)’ setting and a total sample size of *n* = 60 (sample size per arm *n* = 30; 1:1). The effect of sex on study outcomes was determined using the ‘*t* tests—Means: Differences between two independent means (two groups)’ setting of the said program and a total sample size of *n* = 30 (sample size per arm *n* = 15, 1:1). The required effect sizes Cohen *d*_z_ (dependent measures) or Cohen *d* (independent measures) were computed based on each group’s mean (SD) and their correlation coefficient (*d*_z_ only). The other settings were as follows: α = 0.05, two-tailed; power = 0.80. These data are provided in Supplementary Table S1 (dependent comparisons) and Supplementary Table S2 (independent comparisons).

### 2.3. Characteristics of the Study Group

The treatment group consisted of 30 patients (15 female and 15 male) from the Department of Orthodontics and Dentofacial Orthopedics, University Hospital, LMU Munich. All patients were treated by one experienced orthodontist. At the beginning of orthodontic treatment (T1), the patients’ ages ranged between 11.7 and 17.5 years (average 13.4 years). The treatment duration ranged from 1.2 to 5.1 years (average 2.9 years). At the end of orthodontic treatment (T2), the patients’ average age was 16.3 years (14.1–20.6 years). The control group for cephalometric measurements comprised 30 age- and sex-matched patients (15 female and 15 male) from the growth studies at the Universities of Iowa, Oregon, and Denver (‘Craniofacial Growth Legacy Collections Project’ of the AAOF). At T1, the age of the control patients ranged between 11.5 and 17.8 years (average 13.3 years). At T2, the average age of the control patients was 16.2 years (14.0–20.3 years). To verify the inclusion criteria ‘skeletal class I’, the ANB angle was measured using lateral cephalometric radiographs before the start of orthodontic treatment (T1). The average ANB angle was 2.3° ± 1.1° in the study group and 2.5° ± 1.1° in the control group. Further, possible differences between the study and control groups before the start of treatment were evaluated using cephalometric analyses. Comparing the groups, no significant differences were found regarding sagittal parameters (SNA, SNB, and ANB) and vertical parameters (MP–SN, PP–SN, and MP–PP) at T1. Regarding the inclination of the lower incisors, no differences were found between the control and treatment groups (L1–ML) at T1 (95.4° ± 7.3° vs. 95.3° ± 6.7°). The only parameter differing between the study group and the control group was the inclination of the upper teeth. The upper incisors showed a +3.2°/+3.4° greater inclination in the control group compared to the treatment group at T1 (U1–PP: 114.3° ± 5.5° vs. 111.1° ± 5.5°, *p* = 0.001; U1–SN: 106.2° ± 6.3° vs. 102.8° ± 6.4°, *p* = 0.014). Comparing cephalometric parameters of males and females within the control group and within the study group, no significant differences were found at T1.

## 3. Results

### 3.1. Sagittal and Vertical Parameters

**Figure 2:**
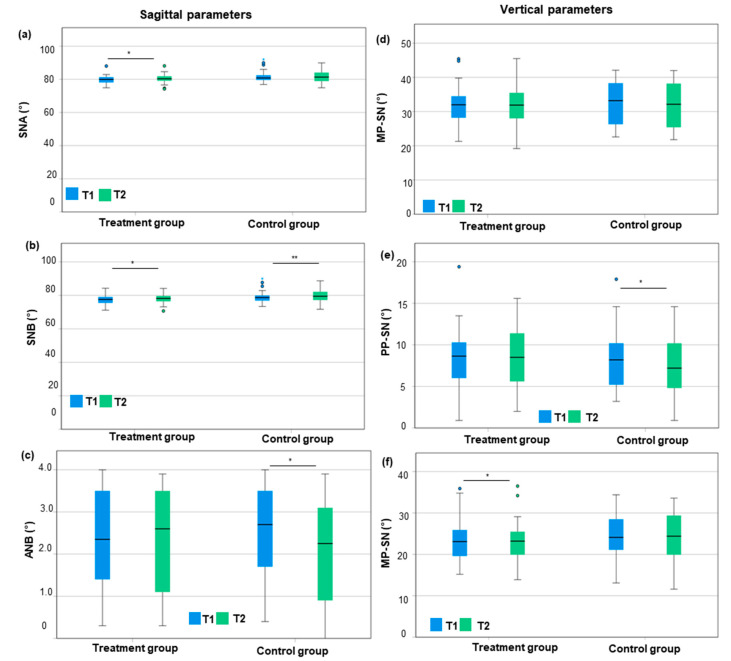
Comparison of sagittal and vertical cephalometric values between T1 and T2 regarding the treatment group and control group using box plots. (**a**–**c**) Sagittal parameters: (**a**) SNA, (**b**) SNB, and (**c**) ANB; (**d**,**e**) vertical parameters: (**d**) mandibularplane (MP)–Sella–Nasion plane (SN), (**e**) palatal plane (PP)–Sella–Nasion plane (SN), and (**f**) MP–SN. T1 = start of orthodontic treatment/matched time point for controls; T2 = end of orthodontic treatment/matched time point for controls. *, *p* < 0.05 = significant; **, *p* ≤ 0.01 = very significant.

With respect to the treatment group, the SNA angle was, on average, 0.5° greater after the orthodontic treatment than before treatment (80.3° ± 2.8° vs. 79.8° ± 2.6°; *p* = 0.034; *d*_z_ = 0.39; power = 0.838) (Figure 2, Supplementary Table S1). The SNB angle was also 0.6° greater after the orthodontic treatment than before (78.0° ± 2.6° vs. 77.4° ± 2.6°; *p* = 0.014; *d*_z_ = 0.50; power = 0.967) (Figure 2, Supplementary Table S1). The ANB angle showed no significant change before vs. after treatment (0.0° ± 0.6°) (Figure 2).

Regarding the control group, the SNA increased slightly from T1 to T2 (81.6° ± 3.8° vs. 81.8° ± 3.7°) (Figure 2). The SNB angle increased significantly by 0.6° (79.1° ± 3.8° vs. 79.7° ± 3.7°; *p* = 0.009; *d*_z_ = 0.49; power = 0.962) (Figure 2, Supplementary Table S1). The ANB angle decreased significantly by 0.4° (2.5° ± 1.1° vs. 2.1° ± 1.3°; *p* = 0.001; *d*_z_ = 0.60; power = 0.996) (Figure 2, Supplementary Table S1). Comparing both groups, the reduction in the ANB angle from T1 to T2 in the control group was significant compared to the treatment group (−0.4° ± 0.6° vs. 0.0° ± 0.6°). This implies that the mesial growth of the mandible was greater in controls compared to the treatment group. Considering the treatment group, a 0.5° smaller MP–PP angle was measured after the orthodontic treatment than before the treatment (23.1° ± 5.2° vs. 23.6° ± 5.2°; *p* = 0.048; *d*_z_ = 0.36; power = 0.779) (Figure 2, Supplementary Table S1). The PP–SN angle remained stable (8.5° ± 3.5° vs. 8.3° ± 3.8°) and the MP–SN angle decreased, but not significantly (31.5° ± 6.2° vs. 31.9° ± 5.9°) (Figure 2). Regarding the control group, a significant reduction in the MP–SN and PP–SN angle by 0.7° and 0.8° from T1 to T2 was found (MP-SN: 32.4° ± 6.4° vs. 31.7° ± 6.5°; *p* = 0.027; *d*_z_ = 0.34; power = 0.734; PP-SN: 8.1° ± 3.6° vs. 7.3° ± 3.5°; *p* = 0.027, *d*_z_ = 0.44; power = 0.916) (Figure 2, Supplementary Table S1). This implies an anterior rotation of the maxillary and mandibular planes by similar amounts. Hence, the MP–PP angle remained stable from T1 to T2 (24.4° ± 5.6° vs. 24.3° ± 5.6°) (Figure 2). Comparing both groups, orthodontic treatment with SL brackets was accompanied by a reduction in the interbase MP–PP angle (−0.5° ± 1.4° vs. +0.0° ± 2.5°; n.s.).

### 3.2. Inclination of the Upper and Lower Incisors

**Figure 3:**
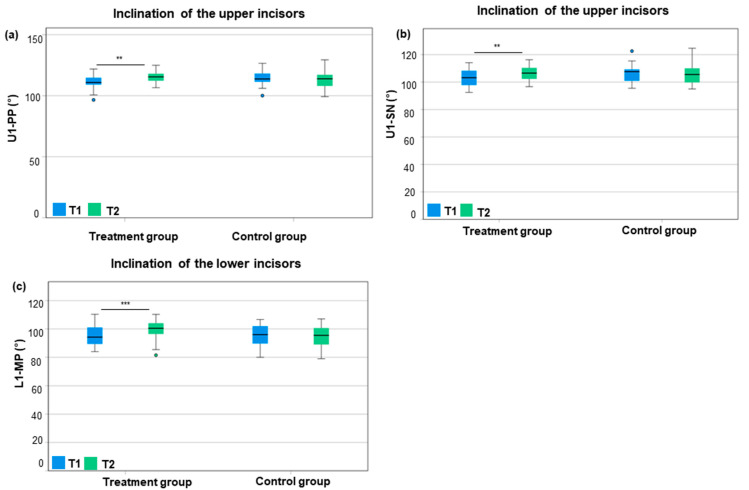
Comparison of inclination of the incisors between T1 and T2 regarding the treatment group and control group using box plots. (**a**,**b**) Inclination of the upper incisors (U1): (**a**) U1–palatal plane (PP), (**b**) U1–(Sella–Nasion line) SN; (**c**) inclination of the lower incisors (L1): L1–MP (mandibular plane). T1 = start of orthodontic treatment/matched time point for controls; T2 = end of orthodontic treatment/matched time point for controls. ** = *p* < 0.01 = very significant; *** = *p* < 0.001 = highly significant.

Considering the treatment group, the inclination of the upper incisors to the palatal plane (U1–PP) increased significantly by +3.8° from T1 to T2 (111.1° ± 5.5° to 114.9° ± 4.4°; *p* = 0.001; *d*_z_ = 0.64; power = 0.998) (Figure 3, Supplementary Table S1). Similarly, the inclination of the upper incisors to the Sella–Nasion plane (U1–SN) showed an increase of +3.7° from T1 to T2 (102.8° ± 6.4° to 106.5° ± 5.4°; *p* = 0.004; *d*_z_ = 0.58; power = 0.992) (Figure 3, Supplementary Table S1). The inclination of the lower incisors to the mandibular plane (L1–MP) was significantly more proclined by +4.4° after orthodontic treatment compared to before (99.7° ± 6.5° vs. 95.3° ± 6.7°; *p* < 0.001; *d*_z_ = 0.93; power = 0.999) (Figure 3, Supplementary Table S1).

Considering the control group, a reclination of −1.4° (U1–PP) and −0.6° (U1–SN) of the upper incisors was seen at T2 compared to T1 (U1–PP: 113.0° ± 6.3° vs. 114.3° ± 5.5°; U1–SN: 105.7° ± 6.9° vs. 106.2° ± 6.3°) (Figure 3). Similarly, the inclination of the lower incisors was −0.4° more reclined at T2 compared to T1 (94.9° ± 7.7° vs. 95.4° vs. 7.3°) (Figure 3). However, differences in the control group were statistically not significant.

The difference in inclination changes in the upper incisors from T1 to T2, i.e., +3.8° ± 6.0° (U1–PP) and +3.7° ± 6.4° (U1–SN) in the treatment group and −1.4° ± 3.8° (U1–PP) and −0.6° ± 3.4° (U1–SN) in the control group, was highly significant (*p* < 0.001; *p* = 0.003). Similarly, the inclination changes in the lower incisors (L1–MP), i.e., 4.4° ± 4.7° in the treatment group and −0.4° ± 2.9° in the control group, were highly significant (*p* < 0.001). Regarding changes per year, the inclination of the upper incisors increased by +1.6° ± 2.3° per year (U1–PP) and +1.5° ± 2.5° per year (U1–SN) in the treatment group and decreased by 0.4° ± 1.3° (U1–PP) and by −0.1° ± 1.1° per year (U1–SN) in the control group (*p* < 0.001; *p* = 0.003). The inclination of the lower incisors (L1–ML) increased by 1.4° ± 1.9° per year in the treatment group and decreased by −0.2° ± 1.4° per year in the control group (*p* < 0.001).

The total average proclination of the upper incisors was +5.2° (+2.0°/year) to the palatal plane and +4.3° (1.6°/year) to the Sella–Nasion plane after orthodontic treatment with SL brackets compared to controls. Comparably, the inclination of the lower incisors increased by +4.8° (1.6°/year) to the mandibular plane after orthodontic treatment with SL brackets compared to controls.

### 3.3. Sex-Specific Characteristics

**Figure 4:**
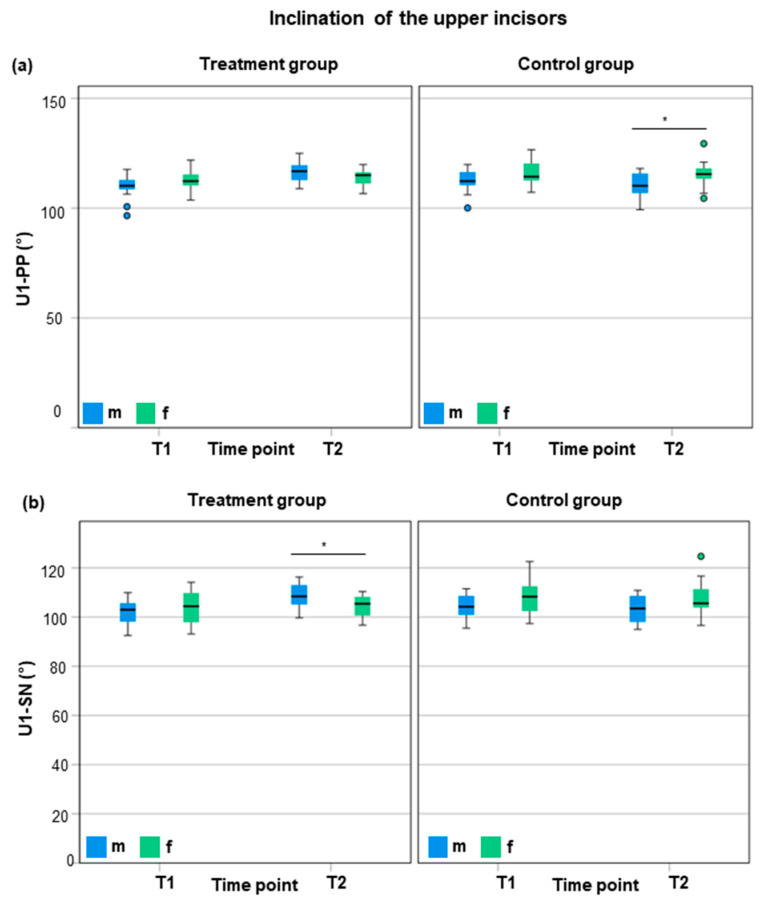
Comparison of cephalometric inclination values of the upper incisors between males and females regarding the treatment group and control group at T1 and T2 using box plots. (**a**) Inclination of the upper incisor to the palatal plane (U1–PP); (**b**) inclination of the upper incisor to the Sella–Nasion line (SN). T1 = start of orthodontic treatment/matched time point for controls; T2 = end of orthodontic treatment/matched time point for controls; m = male; f = female. * = *p* < 0.05 = significant.

Regarding sagittal (SNA, SNB, and ANB) and vertical cephalometric parameters (MP–PP, MP–SN, and PP–SN), as well as the inclination of the lower incisors (L1–MP) at T2, no significant sex-specific differences were found within the treatment or control group. Considering changes between T1 and T2 in the treatment group, the sagittal mandibular growth (ΔSNB) was significantly more pronounced in male compared to female patients (1° ± 1.3° vs. 0.1° ± 0.9°; *p* = 0.049; *d* = 0.80; power = 0.567) (Supplementary Table S2). Similarly, the ΔSNB/year was greater in males compared to females (0.4° ± 0.5°/year vs. 0.0° ± 0.3°/year; *p* = 0.020; *d* = 0.97; power = 0.727) (Supplementary Table S2). Similarly, the sagittal maxillary growth was (not significantly) greater in males (0.9° ± 1.4°; 0.4° ± 0.5°/year) compared to female patients (0.1° ± 1.0°; 0.0° ± 0.4°/year). No sex-specific differences were found regarding the ANB angle (m: 0.0° ± 0.2°; f: 0.0° ± 0.2°). However, significant differences were found with respect to the inclination of the upper incisors and sex in the control and treatment groups at T2 (Figure 4).

In the control group, male patients presented a −5.4° smaller inclination of the upper incisors (U1–PP) compared to females at T2 (110.3° ± 5.6° vs. 115.7° ± 5.8°, *p* = 0.016; *d* = 0.95; power = 0.707) (Figure 4, Supplementary Table S2). Regarding the treatment group, the inclination of the upper front teeth (U1–SN) was +4.3° more proclined in males compared to females at T2 (108.6° ± 5.3° vs. 104.3° ± 4.7° vs., *p* = 0.027; *d* = 0.86; power = 0.622) (Figure 4, Supplementary Table S2).

Considering changes between T1 and T2 in the treatment group, male patients presented a significantly greater proclination of the upper incisors compared to female patients (ΔU1–PP: 6.5° ± 6.1° vs. 1.2° ± 4.7°, *p* = 0.012; *d* = 0.97; power = 0.730) (ΔU1–SN: 6.6° ± 6.1° vs. 0.7° ± 5.3°, *p* = 0.009; *d* = 1.323; power = 0.939) (Supplementary Table S2). With respect to changes per year, the upper incisors proclined by 2.6° ± 2,4° (ΔU1–PP/year) and 2.7° ± 2.5° (ΔU1–SN/year) in male patients and 0.5° ± 1.6° (ΔU1–PP/year) and 0.3° ± 2.0° (ΔU1–SN/year) in female patients. The increase in proclination per year was significantly greater in males than females (ΔU1–PP/year: *p* = 0.011; *d* = 1.03; power = 0.777) (ΔU1–SN/year: *p* = 0.008; *d* = 1.06; power = 0.800) (Supplementary Table S2).

To conclude, male patients of the control group presented a −5.4° greater reclination of the upper incisors (U1–PP) compared to females at T2. Within the treatment group, male patients showed a +4.3° greater proclination of the upper front teeth (U1–SN) compared to females. During orthodontic treatment with SL brackets, the change in proclination of the upper incisors was greater in males compared to females by +5.3° (+2.1°/year) to the palatal plane and +5.9° (2.6°/year) to the Sella–Nasion plane.

## 4. Discussion

Considering cephalometric sagittal measurements, a significant reduction in the ANB angle by −0.4° was seen in the control group compared to the treatment group from T1 to T2. Comparing vertical skeletal changes between T1 and T2, the controls presented an anterior rotation of both jaws by the same amount, resulting in a stable interbase angle, while orthodontic treatment with SL brackets was accompanied by a (non-significant) reduction in the interbase angle of MP–PP by −0.5° ± 1.4°.

Regarding inclination measurements of the upper incisors, cephalometric measurements presented a proclination of the upper incisors by +5.2° to the palatal plane (U1–PP) and +4.3° to the Sella–Nasion plane (U1–SN) after orthodontic treatment with SL brackets compared to controls. In this study, the inclination of the upper incisors was ortho-axial prior to orthodontic treatment with SL brackets (U1–PP: 111.1 ± 5.5; U1–SN: 106.2 ± 6.3), while the control group presented (significantly) more proclined upper incisors at T1 (U1–PP: 114.3 ± 5.5, U1–SN: 111.1 ± 5.5). Accordingly, Hegele et al. [36] demonstrated that orthodontic treatment with SL brackets is accompanied by a proclination of the upper incisors. The proclination effect comprised +3.8° using SL CAD-CAM brackets (Insignia^TM^, Ormco, Orange, CA, USA) and +0.2° using conventional SL brackets (Damon, Ormco, USA) [36]. These differences can partly be explained by less protruded upper incisors before treatment in the group with CAD-CAM brackets (U1–SN: 111.4°) compared to the group with conventional brackets (U1–SN: 114.5°) [36]. At the end of treatment, similar inclination values of the upper incisors were found with conventional (U1–SN: 114.7°) and CAD-CAM brackets (U1–SN: 115.2°) [36]. Notably, it was shown that incisors with proclined inclination prior to treatment present a smaller proclination effect compared to incisors with ortho-axial inclination prior to treatment [35]. In the upper arch, anterior space analyses revealed an average of 0.43 mm ± 1.34 mm, and the Little irregularity index [38] was 3.44 ± 2.04 in the upper arch. As solely minimal irregularities and, on average, slight excess space were detected, proclination because of crowding resolution cannot be expected in this patient cohort. Hence, proclination was solely an effect of vestibular-attached SL brackets.

Considering the inclination measurements of the lower incisors, cephalometric evaluations in this study showed a proclination by +4.8° to the mandibular plane (L1–MP) after orthodontic treatment with SL brackets compared to controls. In accordance, Hegele et al. [36] showed a significant proclination effect on the lower incisors by +3° (L1–MP: 96.4° vs. 99.4°) using SL CAD-CAM brackets (Insignia™, Ormco, USA) and +7° (L1–MP: 94.5° vs. 101.5°) using conventional SL brackets (Damon, Ormco, USA) [36]. Hence, the proclination of the lower incisors was greater in the lower jaw (+7°) compared to the upper jaw (+0.2°) using conventional SL brackets [36]. Similarly, other studies [34,35] showed a proclination of the lower incisors by +3.1° (L1–MP) and +4.4° (L1–MP) after treatment with SL brackets. Another study investigating the inclination of the lower incisors after orthodontic treatment found a proclination of 1.44° ± 7.19° (IMPA = angle between long axis of L1 and MP~L1–MP) [43]. Correlation analyses revealed that patients with inclined incisors after treatment (IMPA > 95°) showed changes in clinical crown height and gingival scallop compared to patients with ortho-axial inclination of the lower incisors (IMPA < 95°) [43]. Anterior space analyses revealed an average of −0.01 ± 0.54 mm in the lower arch. The Little irregularity index [38] revealed mild crowding of 2.32 ± 1.60 in the lower arch. The impact of proclination to resolve crowding could have only had a minimal impact on lower incisor proclination in this patient cohort. Our results are in accordance with previous studies [34,35,36], presenting a significant proclination of the lower incisors after orthodontic treatment with SL brackets. As it was shown that proclination leads to an apical migration of the gingiva [43] and that vestibular tooth movements correlate with an altered vestibular gingival contour [44], severe proclination of the incisors should be avoided via modification to the treatment strategy.

Further, we detected sex-specific differences regarding incisor inclination. Males presented a higher protrusion of the upper incisors by +5.3° (U1–PP) and +5.9° (U1–SN) compared to females during orthodontic treatment with SL brackets. In the medical field, it has been proposed to include sex in treatment planning to enable individualized ‘precision’ medicine [45]. Notably, sex and gender can have a favorable or unfavorable impact on the treatment prognosis of the disease [46]. Considering orofacial orthopedics, growth-related sex differences are well-known in treatment planning with removable or fixed functional appliances [47]. The treatment efficiency considering skeletal parameters was shown to be greater in male patients during the peak of growth compared to early treatments, while those effects were less pronounced in female patients [48]. Considering orthodontic treatment with different bracket systems, the impact of sex and age on molar angulation and mandibular growth was investigated [49]. In male patients, the linear mandibular growth was greater in males, which alleviated the treatment of class II malocclusion [49].

The strength of this study is the methodology, as previous studies have lacked a comparison with untreated controls or used a control group treated with a different bracket prescription, e.g., Damon vs. Roth prescription [37]. Considering the study population, cephalometric measurements were compared between a treatment group (orthodontic treatment with SL brackets) and an age- and gender-matched control group at two time points, i.e., before treatment (T1) and after treatment (T2), and matched time points for controls. The male to female ratio was 1:1 in both groups (15 male, 15 female), the average duration of treatment was 2.9 ± 1.0 years, and the time interval between the X-rays (T1–T2) in the control group was 2.9 ± 1.1 years, accordingly. One of the inclusion criteria was that patients were treated using SL brackets (0.022″ slot, MBT prescription). Although the practitioner was not selected in advance due to the retrospective study design, a similar treatment concept was ensured by the inclusion of patients treated in the Department of Orthodontics and Orofacial Orthopedics by one experienced orthodontist. The comparison of the groups presented no significant differences between the study and control groups, except for a greater inclination of +3.3° of the upper incisors in the control group at T1. More reclined upper incisors in the treatment group might be explained by pretreatment with functional orthodontic appliances (two-phase concept), leading to a reclination of the upper incisors in the case of class-II appliances [50,51]. Furthermore, no significant differences were seen between males and females within the control or study group at T1. In this study, lateral cephalometric radiographs were recorded digitally and analyzed digitally. The reproducibility of digitally analyzed lateral cephalometric radiographs has been proven [52,53,54,55,56]. Furthermore, digital evaluation of digitally recorded lateral cephalometric radiographs had the highest reproducibility compared to manually recorded or digitized analogue recorded lateral cephalometric radiographs [57]. The validity and reproducibility of cephalometric analyses have been demonstrated. As placement of anatomical points by different examiners is a potential source of error [58,59], all cephalometric measurements were performed by a single examiner and intra-rater accuracy was evaluated. The relative technical error of the mean (rTEM) was less than 5% for all values except the ANB angle (accepted as rTEM < 15%). The higher deviation value can be explained by the small numerical value (single-digit, ANB 0–4°). Anterior tooth axes are the most difficult to determine and have the lowest reproducibility [60]. Yet, the rTEM for repeated measurements was 0.50° for U1–PP and U1–SN and 0.43° for L1–MP. To summarize, the control group selected for this study was appropriate for statistical comparisons. Further, homogeneity was ensured within the study group as all patients were treated by one experienced orthodontist using the same treatment concept. Moreover, digital analyses of lateral cephalograms showed a small rTEM for the measurement of incisor inclination.

The limitation of the study is the retrospective design, leading to potential weaknesses of the investigation [61], e.g., due to the retrospective nature of the study, the treatment concept was not determined in advance. A retrospective study design was chosen as we aspired to investigate growth-related changes via comparisons with a non-treated control group. A prospective study design including lateral cephalometric radiographs in untreated patients would not be possible in Germany due to ethical restrictions. However, comparability of the study group is given as all patients were treated by the same orthodontist using the same archwire sequence and treatment concept. Another limitation of the study is the high rTEM of 13.94% found for the ANB angle. The variable ‘ANB angle’ is a very small value as it ranges between 0° and 4° in skeletal class I patients. The ANB angle was already measured for all patients for diagnosis and treatment planning before and after orthodontic treatment in the Department of Orthodontics and Dentofacial Orthopedics, University Hospital, LMU Munich. All patients included in this study were already diagnosed as ‘skeletal class I’ patients. For verification of the inclusion criteria ‘skeletal class I’, the ANB angle was measured using lateral cephalometric radiographs before the start of orthodontic treatment at T1. An average ANB angle of 2.3° with a standard deviation of 1.1° was found in the study group. The average ANB angle was 2.5° with a standard deviation of 1.1° in the control group. The high rTEM of 13.94% did not change the diagnosis ‘skeletal class I’. Further, a standard deviation of 1.1° is very small. While intra-examiner reliability was performed, no inter-examiner reliability was investigated in this study, as all lateral cephalograms were investigated by one examiner. While the validity, reproducibility, and reliability of digitally analyzed lateral cephalometric radiographs have been proven [52,53,54,55,56,57,62], the placement of anatomical points by different examiners is a potential source of error [58,59]. Notably, the inter-examiner tracing error regarding the setting of anatomical points was higher than the intra-examiner tracing error [59]. Hence, the study design involved only one examiner who investigated all lateral cephalograms to exclude this potential source of error. A further limitation is that the control group was generated using lateral cephalograms of orthodontically untreated girls and boys from growth studies at the Universities of Iowa, Oregon, and Denver, which are part of the ‘Craniofacial Growth Legacy Collections Project’ [40]. This might lead to potential ethnical and geographical differences as the study group was collected in Germany, Europe. Orthodontists are trained to use established norms and values that do not include ethnicity [63]. However, ethnic considerations should be included in treatment planning [63]. One study geographically performed in the U.S. revealed that the majority (60.8%) of the orthodontic patients are Non-Hispanic Whites [64]. Further, another study comparing orthodontic therapies regarding different ethnicities showed that the prevalence of non-extraction cases using fixed orthodontic appliances is higher among Americans (80–85% in Connecticut) and Europeans (~90% in Germany) as Caucasian jaws are wider and longer compared to Asians [65]. In contrast, Asians and Iranians require the extraction of premolars more often (60% in Iran) [65]. In this study, the collection of lateral cephalograms of untreated patients in Germany was not possible due to ethical restrictions. Similarity of the study and control groups was assured by matching the controls according to age and sex. As the control group in this study was from the U.S., the ethnic and geographical differences were minimized.

The clinical implications are as follows: (I) The application of passive SL brackets was accompanied by moderate protrusion of the upper incisors and severe protrusion of the lower incisors. Orthodontists must consider, according to initial diagnostics, whether protrusion of the incisors is clinically acceptable. Slight protrusion of the upper incisors can be necessary in compensated class III patients. However, protrusion of the lower incisors can lead to an anterior crossbite in class III patients. Hence, the application of SL brackets should be avoided for lower incisors in class III patients, and standard edgewise brackets should be used instead. Considering class II patients, protrusion of the upper incisors increases the overjet. Further, protrusion of the lower incisors in class II patients can hinder the correction of a distal occlusion. Therefore, SL brackets should only be applied carefully in class II patients, and anchorage should be planned accordingly. Protrusion of the lower incisors is an undesired side effect of orthodontic treatment with SL brackets. Severe protrusion of the lower incisors can lead to anterior recessions and unstable treatment results. (II) To the best of our knowledge, sex-specific characteristics regarding incisor inclination have not been investigated thus far. We have shown that proclination was more enhanced in males compared to females.

The future directions of study that practitioners must consider include the fact that male patients present a higher risk of incisor proclination compared to females. Eventually, the treatment concept must be adapted according to sex and gender in the future. Our results are highly relevant as they pave the way for future studies focusing on sex- and gender-specific orthodontics and ‘precision’ orthodontics. Future studies should focus on other possible sex- and gender-related differences regarding the treatment outcome. Possible sex- and gender-related differences, e.g., the bone metabolism, immunity, or microbiome of the patients, that could impact tooth movement should be investigated in future trials.

## 5. Conclusions

This study revealed a clinically relevant protrusion effect during orthodontic treatment with SL brackets of +5.2°/+4.3° (U1–PP/U1–SN) for the upper incisors and +4.8° (L1–MP) for the lower incisors compared to controls. The practitioner must decide to what extent these protrusion effects are desirable or not regarding the initial cephalometric findings and other clinical parameters, like the gingival margin. Further, sex-specific differences were found, with males showing a greater proclination of the upper incisors by +5.3° (U1–PP) and +5.9° (U1–SN) compared to females in the treatment group. In the future, orthodontic treatment planning should also include sex- and gender-related differences regarding the treatment outcome. Biomechanical treatment concepts must be planned accordingly. Reverse torque for the lower incisors or increased anchorage should be applied in males during orthodontic treatment. As greater proclination was found in males, it is likely that other tooth movements are also enhanced in males. Sex- and gender-specific differences might be explained by alterations in osteoimmunology, immunity, microbiome, or hormones. Future prospective clinical studies should include sex- and gender-specific evaluations to pave the way for precision orthodontics regarding the individual’s biology.

## Data Availability

Data are unavailable due to privacy and ethical restrictions.

## Supplementary Materials

The following supporting information can be downloaded at: https://www.mdpi.com/article/10.3390/jcm14103370/s1, Table S1: Post-Hoc Power Analysis for paired-samples *t* test; Table S2: Post-Hoc Power-Analyse für independend samples *t* test.
