# Supplementary material for: Inclination Changes in Incisors During Orthodontic Treatment with Passive Self-Ligating Brackets
Source: J Clin Med. 2025 May 12;14(10):3370. doi: 10.3390/jcm14103370 (PMC12112268; doi:10.3390/jcm14103370)
Supplement: Supplementary file 1 [file jcm-14-03370-s001.zip › jcm-3570846-supplementary.pdf]

Supplementary Table S1: Post-Hoc Power Analysis for paired-samples t test

Program: G*Power 3.1.9.6 (Mac) (test family: t tests; Statistical test: means: Difference between two dependent means (matched pairs); Type of power analysis: Post hoc)
Two tails; α = 0.05; total sample size = 60 (1:1)

Variable	Control	Control T1	Control T2	Treatment	Treatment T1	Treatment T2	*Corr. between groups*	*p*-value	Effect size d_z	Power
∠ U1-PP (°)		114.3 ± 5.5			111.1 ± 5.5		.593	.001	0.6448743	0.9984237
∠ U1-SN (°)		106.2 ± 6.3			102.8 ± 6.4		.335	.014	0.4642505	0.9425995
∠ SNA (°)					79.8 ± 2.6	80.3 ± 2.8	.888	.034	0.3868282	0.8382491
∠ SNB (°)					77.4 ± 2.6	78.0 ± 2.6	.893	.014	0.4988512	0.9671521
∠ SNB (°)		79.1 ± 3.8	79.7 ± 3.7				.947	.009	0.4898392	0.9617911
∠ ANB (°)		2.1 ± 1.3	2.5 ± 1.1				.861	.001	0.6047155	0.9959356
ΔANB ∠ (°)	-0.4 ± 0.6			-0.04 ± 0.58			-.092	.031	0.412831	0.8820595
∠ MP-PP (°)					23.6 ± 5.2	23.1 ± 5.2	.964	.048	0.3583442	0.77944463
∠ MP-SN (°)		32.4 ± 6.4	31.7 ± 6.5				.949	.027	0.3394221	0.7344054
∠ PP-SN (°)		8.1 ± 3.6	7.3 ± 3.5				.868	.027	0.4379766	0.9157233
∠ U1-PP (°)					111.1 ± 5.5	114.9 ± 4.4	.292	.001	0.6379827	0.9981341
∠ U1-SN (°)					102.8 ± 6.4	106.5 ± 5.4	.416	.004	0.5752804	0.9922936
∠ L1-MP (°)					95.3 ± 6.7	99.7 ± 6.5	.746	<.001	0.9346187	0.999999
ΔU1-PP∠ (°)	-1.4 ± 3.8			3.8 ± 6.0			-.147	<.001	0.6878933	0.9994810
ΔU1-SN∠ (°)	-0.6 ± 3.4			3.7 ± 6.4			.068	.003	0.6108014	0.9964595
ΔL1-MP∠ (°)	-0.4 ± 2.9			4.4 ± 4.7			.149	<.001	0.9335225	0.999999
ΔU1-PP/yr (°/yr)	0.4 ± 1.3			1.6 ± 2.3			-.120	<.001	0.4325174	0.9091089
ΔU1-SN/yr (°/yr)	-0.1 ± 1.1			1.5 ± 2.5			-.011	.003	0.5834406	0.9935164
ΔL1-MP/yr (°/yr)	-0.2 ± 1.4			1.4 ± 1.9			.011	<.001	0.6815314	0.9993844

Supplementary Table S2: Post-Hoc Power-Analyse für independend samples t test

Program: G*Power 3.1.9.6 (Mac) (test family: t tests; Statistical test: means: Difference between two independent means (two pairs); Type of power analysis: Post hoc)

N(male) = N(female) = 15; α = 0.05

Variable	Treatment (males)	Treatment (females)	Control (males)	Control (females)	*P* value	Effect size d	Power
ΔSNB	1 ± 1.3	0.1 ± 0.9			0.049	0.8049845	0.5669686
ΔSNB/yr	0.4 ± 0.5	0.0 ± 0.3			0.020	0.9701425	0.7273672
U1-PP: T2			110.3 ± 5.6	115.7 ± 5.8	0.016	0.9472227	0.7068964
U1-SN: T2	108.6 ± 5.3	104.3 ± 4.7			0.027	0.8584562	0.6216802
ΔU1-PP	6.5 ± 6.1	1.2 ± 4.7			0.012	0.9733376	0.7301635
ΔU1-SN	6.6 ± 6.1	0.7 ± 5.3			0.009	1.328276	0.9394660
ΔU1-PP/yr	2.6 ± 2.4	0.5 ± 1.6			0.011	1.02961	0.7769420
ΔU1-SN/yr	2.7 ± 2.5	0.3 ± 2.0			0.008	1.060143	0.8002538
